# The role of physical education in achieving the sustainable development goals

**DOI:** 10.3389/fspor.2026.1736485

**Published:** 2026-02-25

**Authors:** Ana Žnidarec Čučković, Berislav Ćosić, Dean Dudley

**Affiliations:** 1 Faculty of Kinesiology, University of Zagreb, Zagreb, Croatia; 2 School of Human Movement, University of Queensland, St Lucia, QLD, Australia

**Keywords:** academic workload, curriculum, holistic benefits, infrastructure, physical activity, teacher training, well-being

## Abstract

*The United Nations' Sustainable Development Goals (SDGs)*, adopted by all United Nations member states in 2015, are 17 interrelated objectives designed to promote a sustainable future for people and the planet. These goals are operationalized through 169 specific targets, 25 of which this paper demonstrates can be meaningfully integrated into physical education (PE) teaching practice. Models-based practices (MBPs), such as Cooperative Learning, Sport Education, and Teaching Personal and Social Responsibility, offer effective pedagogical frameworks for advancing the SDGs within PE. The paper discusses these existing models and emerging alternatives that broaden the pedagogical repertoire available to educators. Furthermore, this paper identifies key challenges in the discipline and proposes solutions, including curriculum reform, a reorientation toward transformative learning perspectives, and a reconceptualization of health and well-being. The crucial role of universities and service-learning initiatives is emphasized in preparing future PE teachers to align their practice with the SDGs. Finally, the analysis highlights persistent global and local barriers to implementation, such as inadequate infrastructure, heavy academic workloads, and limited recognition of the holistic benefits of PE and physical activity.

## 1 Introduction

The adoption of the 2030 Agenda for Sustainable Development by the United Nations (UN) in 2015 was driven by the urgent need to address global concerns and ensure prosperity for all. This ambitious agenda aims to establish a sustainable and just society through 17 global goals and 169 specific targets, requiring immediate action from all countries across areas ranging from combating poverty and hunger to addressing health, education, gender equality, climate change, and sustainable cities (1). The goals also integrate sociological, economic, and ethical dimensions to align human development with the future of the planet (2).

Despite the 15-year implementation timeline (2015–2030), some studies indicate that the pace of achieving the goals is not as fast as expected (3), underscoring the critical need for coordinated action across all state and non-governmental institutions (4).

Education is of paramount importance in this endeavor, as it facilitates the acquisition of the knowledge, skills, and attitudes necessary to create a sustainable world (5). The significance of mainstreaming sustainability into educational systems, a focus previously highlighted during the UN Decade of Education for Sustainable Development (2005–2014), is recognized as foundational for promoting widespread change (6). Scholars contend that education plays a vital role in resolving sociological and environmental obstacles (7), a sentiment echoed by its recognition as a key factor in securing the acceptance of the Millennium Development Goals (4).

The potential contribution of physical education (PE) is evident in the importance UNESCO itself places on PE teaching. The 2015 International Charter on Physical Education, Physical Activity, and Sport reaffirmed a 40-year commitment by the UN to sport and PE being a fundamental human right (8). The 2030 Agenda further acknowledges sport as a critical driver for sustainable development and peace through its ability to foster social sensitivity, combat discrimination, and promote health, education, and social inclusion (9).

However, a critical gap exists: analyses of UN documents regarding sustainability in education often fail to mention PE teaching (5). This omission is a risk, as it may prevent relevant bodies from recognizing and leveraging the discipline's potential benefits. This exclusion is especially concerning given the widespread public health challenge of physical inactivity, with 80% of adolescents and young people failing to perform the minimum recommended amount of physical activity, contributing to a worrying increase in sedentary lifestyles (10).

Therefore, this paper addresses this oversight by:

a) identifying the specific global and detailed targets within the 2030 Agenda for Sustainable Development that can be achieved through PE.
b) presenting the necessary characteristics for a renewed approach to PE curriculum and teacher education that will lead to the achievement of these goals.

## 2 Global sustainable development goals

### 2.1 Education for sustainable development (ESD)

Education for Sustainable Development (ESD) is a core strategy for achieving the 2030 Agenda. It focuses on integrating knowledge, attitudes, and skills related to sustainability into educational systems to promote the creation of a sustainable, inclusive, and just society. This approach emphasizes four key areas: reorienting existing curricula, raising public awareness, ensuring comprehensive training across all sectors, and prioritizing quality education (11).

Quality education mandates a holistic approach that nurtures creativity and socioemotional well-being. Key to this is equity and inclusiveness, which requires providing every learner with access to high-quality education and actively addressing systemic barriers such as poverty, gender inequality, and the inclusion of people with disabilities (12). Embracing diversity and accommodating individual learning styles are crucial for creating an equitable environment (13). Furthermore, quality education focuses on developing 21st-century skills, including critical thinking, problem-solving, and collaboration, which are essential for navigating the modern world and preparing students for the labor market, enhanced by the effective integration of technology (14).

Beyond UNESCO's call to action (1), other major organizations have recognized the power of education, and specifically physical activity, in achieving the SDGs. The World Health Organization (WHO) has explicitly linked physical activity to 13 of the 17 SDGs, highlighting its positive health impact alongside co-benefits such as reducing pollution and improving safety. The WHO stresses that encouraging and designing policies to promote active transportation, recreational facilities, and sports participation can significantly contribute to achieving the 2030 Agenda goals (15–17).

Further supporting this perspective, the OECD launched “The Future of Education and Skills 2030” project in 2019 (18) to identify the competencies students need in the modern world. Through its analysis of national PE curricula and practices, the OECD concluded that a quality-oriented, dynamic, and inclusive PE curriculum must center on student well-being. This necessitates adopting a broader, long-term educational view for PE that emphasizes social and emotional skills, student experiences, cognitive development, autonomy, and academic performance (19, 20). This comprehensive shift ensures that physical education is understood as a vital component of holistic student development (21).

### 2.2 Overview of the global sustainable development goals

The 2030 Agenda for Sustainable Development, adopted by all UN member countries in 2015, established 17 global goals and 169 specific targets to be achieved by 2030 (22). This comprehensive framework is the result of an international consultative process involving civil society, governments, and the private sector, ensuring its global applicability (1). The goals cover a wide range of issues, including eradicating poverty, fighting inequality, improving education and health, and preserving the environment. They serve a practical role in shaping global policies and strategies, and as a yardstick to assess progress in achieving sustainability (23).

The goals cover four main dimensions of sustainability:

a) Social Well-being: Goal 1: No Poverty, Goal 2: Zero Hunger, Goal 3: Good Health and Well-being, Goal 4: Quality Education, Goal 5: Gender Equality, and Goal 10: Reduced Inequalities.
b) Economic Growth: Goal 8: Decent Work and Economic Growth, and Goal 9: Industry, Innovation, and Infrastructure.
c) Environmental Preservation**:** Goal 6: Clean Water and Sanitation, Goal 7: Affordable and Clean Energy, Goal 11: Sustainable Cities and Communities, Goal 12: Responsible Consumption and Production, Goal 13: Climate Action, Goal 14: Life Below Water, and Goal 15: Life on Land.
d) Governance and Partnership: Goal 16: Peace, Justice, and Strong Institutions, and Goal 17: Partnerships for the Goals (1).

Although the global Sustainable Development Goals represent an ambitious vision, their implementation faces numerous challenges. Financial resources, political will, global inequalities and disparities in the capacities of different countries to achieve the goals are key factors affecting the effectiveness of implementation. Therefore, an international framework for cooperation is important, as is the engagement of all stakeholders, including the private sector, civil society organizations and local communities, to ensure real change on the ground (23).

## 3 The role of physical education in achieving the global goals of sustainable development

### 3.1 Overview of institutional research

Institutional research has validated the link between sport, physical activity, and the SDGs. At the Sixth International Conference of Ministers and Senior Officials in Charge of physical education and Sports (MINEPS VI), three broad areas of intervention were established: maximizing sport's contribution to sustainable development and peace, protecting the integrity of sport, and developing an inclusive vision for access to physical activity (5, 24, July 14–15). Concurrently, *The Commonwealth Secretariat developed a pivotal study—providing practical indicators to evaluate sport's contribution to the 2030 Agenda—which is significant because it offers an operational framework that policymakers and practitioners can use to monitor and compare progress across contexts* (24, 25).

In 2019, the Ibero-American Sports Council (IBC) identified that while not all goals are equally applicable, sport has a direct connection to 8 of the 17 global goals and 19 specific targets (26). Solidifying these findings, the World Health Organization (WHO) confirmed that 13 of the 17 global goals can be achieved through sport (24). While these reports focus primarily on sport, PE offers a unique pedagogical space to address various global challenges (27) (Table 1).

**Table 1:** Sustainable development goals achievable through physical education and health education classes.

Global goals	Specific goals
Goal 3. Health and Well-being	3.4 Reduce premature mortality and promote mental health and well-being 3.5 Reduce substance abuse 3.6 Reduce Road traffic injuries 3.7 Ensure universal access to sexual and reproductive health services
Goal 4. Quality education	4.1 Ensure that all girls and boys complete primary and secondary education, which should be free, equitable and of good quality 4.4 Improve skills for access to employment, decent work and entrepreneurship 4.5 Reduce gender gaps in education and ensure equality for vulnerable groups 4.7 Improve knowledge to promote sustainable development (e.g., sustainable lifestyles) 4.a Improve school facilities
Goal 5. Gender Equality	5.1 Eliminate discrimination against all women and girls 5.2 Eliminate all forms of violence against all women and girls in the public and private spheres 5.5 Inclusion of women and equal opportunities 5.c Promote gender equality and the empowerment of women and girls
Goal 8. Decent work and economic growth	8.3 Entrepreneurship, creativity and innovation and promoting the formalization and growth of enterprises 8.9 Promote sustainable tourism that creates jobs and promotes local culture and products
Goal 10. Reduced inequalities	10.2 Social, economic and political inclusion of all people 10.3 Ensure equal opportunities and reduce inequality of outcomes regarding income
Goal 11. Sustainable cities and communities	11.6 Reduce the per capita adverse environmental impact of cities, paying particular attention to air quality and the management of municipal and other waste
Goal 12. Responsible consumption and production	12.1 Sustainable consumption and production 12.2 Sustainable management and efficient use of natural resources 12.5 Significantly reduce waste generation 12.8 Ensure information and knowledge relevant to sustainable development
Goal 13. Response to climate change	13.1 Strengthen the adaptive capacity to climate risks and natural disasters in all countries 13.3 Enhance education, awareness-raising and human and institutional capacities to mitigate the impacts of climate change
Goal 16. Peace and justice/strong institutions	16.7 Ensure effective, inclusive, participatory and representative decision-making at all levels

### 3.2 Global goals achievable through physical education classes

Physical education is not considered to directly address Goal 1: No Poverty or Goal 2: Zero Hunger in the available literature (24). The achievable targets start with Goal 3.

#### 3.2.1 Physical education's contribution to health and well-being (goal 3)

Physical education has a direct link to health-related targets, most significantly through the established positive impact of continuous, systematic physical activity on students' mental and psychological states, directly addressing Target 3.4 (Reduce premature mortality and promote mental health and well-being) (28). Furthermore, PE instils values that actively deter drug use, linking it to Target 3.5 (Reduce drug abuse) (29).

The subject also provides an indirect link to other health targets. By contributing to students' understanding of their bodies from a biological perspective, PE can support Target 3.7 (Sexual and reproductive healthcare) (30). *When road-safety education is integrated into the curriculum (e.g., safe movement on roadways, including cycling and other forms of active transportation), PE also links to Target 3.6 (Reduce road traffic injuries)* (24).

#### 3.2.2 Fostering quality education and skills (goal 4)

Physical education is a fundamental right and an indispensable part of a quality education system, establishing a direct link to Target 4.1 (Free and quality education) (8, 24). Neuroscientific evidence strongly links physical activity to improved academic and cognitive performance, including enhanced concentration, better executive function, and increased feelings of well-being (31–34).

Moreover, PE fosters essential skills for employment, such as cooperation, responsibility, and respect, aligning with Target 4.4 (Skills for employment) (10). As an ideal setting for co-education and teamwork, PE also helps reduce gender gaps and ensures equality for vulnerable groups, directly supporting Target 4.5 (Equality) (24, 35). PE's sustainability link addresses Target 4.7 (Sustainability knowledge and skills) by teaching principles through physical activity in the natural environment and presenting sustainable alternatives (24, 36). *The International Olympic Committee (IOC) noted a two-way connection with this objective: (1) PE can advance sustainability competencies (Target 4.7) through structured learning experiences; and (2) education for sustainable development can, in turn, strengthen PE quality by legitimising broader learning outcomes beyond fitness and sport performance* (26).

#### 3.2.3 Promoting gender equality (goal 5) and reducing inequalities (goal 10)

Physical education has a direct link to gender equality by offering opportunities to create a non-discriminatory environment that encourages women's inclusion and empowerment (5, 26). This directly addresses Targets 5.1, 5.2 (End discrimination and violence against women), and 5.5 (Equality of opportunity) (24). The empowerment of women (Target 5.c) can also be achieved by introducing appealing sports initiatives in PE, ideally supported by external institutions (24).

Similarly, sport is an opportunity to combat all forms of discrimination (26). PE promotes cooperative attitudes and **inclusion** (37), establishing a direct link to Goal 10: Reduced Inequalities, specifically supporting Targets 10.2 (Inclusion of all people) and 10.3 (Ensure equal opportunities) (24).

#### 3.2.4 Economic growth and sustainable development

Physical education establishes an indirect link to Goal 8: Decent Work and Economic Growth by cultivating valuable transferable values for employment, such as cooperation, tolerance, and emotional control (38). Target 8.3 (Entrepreneurship and innovation) is linked by using models like Sport Education to encourage careers in the appealing sports sector (24, 26). Furthermore, promoting traditional sports, local games, and cultural customs in PE supports cultural heritage and local tourism, addressing Target 8.9 (Sustainable tourism).

Goal 11: Sustainable Cities and Communities has a pedagogical link through a Community Service Learning framework, allowing PE to engage with Targets 11.3 (Sustainable urbanization) and 11.7 (Access to green and public spaces), for example, by maintaining a local exercise park (24, 26). The subject also addresses Target 11.6 (Reduce negative environmental impact of cities) by encouraging sustainable modes of transport, such as walking or cycling.

Goal 12: Responsible Consumption and Production has an educational link as PE provides a practical context for promoting sustainable consumption habits (Targets 12.2, 12.5, and 12.8) (5). *This can be addressed through authentic PE tasks such as auditing equipment use and waste during units, designing low-cost activity stations with reused materials (with documented safety checks), and evaluating the environmental impact of sport or school events as part of active learning projects.*

A direct link to Goal 13: Climate Action is established through PE's impact on environmental protection, aligning with Target 13.3 (Education and awareness) (24). Participating in activities like “plogging” (running while collecting waste) positively impacts students' environmental thinking and attitudes (39).

#### 3.2.5 Peace, justice, and partnerships

Sport and physical activity promote tolerance, equality, and cooperation (26), providing a pedagogical link to Goal 16: Peace, Justice, and Strong Institutions. PE can improve interpersonal relationships and link to Target 16.7 (Inclusive decision-making) (24). This goal is also connected to the Teaching Personal and Social Responsibility (TPSR) model, an increasing used and valid pedagogical model for teaching civic values through PE (40).

Finally, PE contributes to Goal 17: Partnerships for the Goals by fostering emotional connections and cooperation among stakeholders (26), thereby aligning with the need for partnership and coordination (1).

### 3.3 Models of implementation of physical education teaching and global sustainable development goals

Physical education, when strategically integrated with the Sustainable Development Goals, offers a robust framework for fostering holistic development beyond mere physical prowess (41). In addition to identifying the goals of the 2030 Agenda that can be achieved through PE, it is also necessary to link specific goals to different models of PE teaching implementation that are based on practice. This step will allow us to more clearly define what teaching aimed at achieving the goals should look like. PE is characterized by great methodological diversity, which is a consequence of the characteristics of the content itself that is taught. Thanks to this, several teaching methods used in PE have developed. Some of the models that are applicable in PE to achieve global goals are collaborative learning, t*he Sport Education Model and the Teaching Personal and Social Responsibility (TPSR) model.* In addition, newer models such as adventure learning, health education, or independent production of teaching aids are increasingly present (24). *To strengthen alignment with current PK–12 trends, additional curriculum models and instructional frameworks may also be considered, such as Inquiry-based Learning, Universal Design for Learning (UDL), and Restorative Practices.*

#### 3.3.1 Cooperative learning

Cooperative learning involves organizing students into heterogeneous groups based on their differing levels of knowledge and ability. Within these groups, students are not only responsible for mastering the learning content but also for supporting the progress of their peers (42). A growing body of literature highlights the value of cooperative learning in physical education (PE). Research indicates that this approach enhances students' engagement in physical activity, reduces anxiety related to performance, and strengthens confidence in their physical capabilities (43).

The cooperative learning strategy rests on three core principles: positive interdependence, individual and group responsibility, and promotive interaction (44, 45). Positive interdependence describes the perception that group members' success is mutually beneficial—each student's achievement contributes to the collective outcome. This interdependence is fostered when learners take ownership of their own actions, engage actively with the content, and complete assigned tasks conscientiously (46–48).

Individual responsibility refers to each learner's duty to participate meaningfully in the group's work: sharing ideas, listening to others, and preparing for collaborative activities. Students are expected to take responsibility for their assigned roles, assist peers when possible, and seek help when needed. This element ensures maximal engagement and contribution from all participants (49, as cited in (91)). Group responsibility, in turn, encompasses the shared commitment to achieving common objectives through cooperation, mutual support, joint decision-making, and collective accountability for outcomes (46, 47).

Although cooperative learning is not unique to PE, the inherently interactive and participatory nature of physical education amplifies its benefits (50). The approach can contribute directly to the advancement of several Sustainable Development Goals (SDGs). For instance, it aligns with Goal 16.7, which emphasizes inclusive, participatory, and representative decision-making—features that mirror the cooperative learning process (24, 50). It also relates to Goal 4.4, which promotes the development of entrepreneurial and collaborative competences relevant to employability, as cooperative learning nurtures innovation, teamwork, and problem-solving (24, 51). Moreover, the model supports Goal 4.5 by fostering inclusion and respect for diverse perspectives, thus enhancing participation among vulnerable groups (52). Finally, cooperative learning connects to Goal 8.3, which focuses on encouraging entrepreneurship, creativity, and innovation—capacities strengthened through the social and interpersonal skills cultivated in PE (53).

#### 3.3.2 Model of teaching personal and social responsibility

The Teaching Personal and Social Responsibility (TPSR) (54) model has been widely validated as an effective pedagogical framework in physical education (PE) for fostering students' competencies related to responsibility and ethical behaviour (40). This model promotes fundamental social values such as respect, equality, and fairness (37). Within PE, TPSR plays a pivotal role in cultivating both personal and social dimensions of responsibility that extend beyond the gymnasium and into everyday life.

Personal responsibility involves an individual's awareness of their own health and wellbeing, and the capacity to make informed, autonomous decisions about physical activity, nutrition, and lifestyle (55). Social responsibility, in contrast, concerns understanding how one's actions affect others and the environment, and actively contributing to the health and wellbeing of the broader community (54). PE offers a unique context for developing these dual forms of responsibility because it integrates physical, social, and moral learning within a shared activity setting.

Through participation in varied physical activities, students learn the importance of physical activity for health, acquire the skills needed to engage in sport and recreation, and develop knowledge about nutrition and healthy living (56). At the same time, PE provides a setting for practising key social skills such as teamwork, cooperation, empathy, fair play, and respect for others. All of which are core elements of social responsibility (57). By embedding TPSR within PE, teachers can guide students to become self-regulated, socially aware individuals who recognise their role in promoting collective wellbeing.

Importantly, the TPSR model contributes to advancing several Sustainable Development Goals (SDGs) associated with equality and inclusion. It aligns closely with Goal 5 (Gender Equality)—particularly sub-goals 5.1, 5.2, 5.5, and 5.c which aim to eliminate discrimination and violence against women, ensure equal participation in decision-making, and empower women and girls (58). By promoting respect and equitable participation, TPSR fosters an inclusive learning climate consistent with these global objectives.

The model also aligns with Goal 16.7, which advocates for inclusive and participatory decision-making, by empowering students to take responsibility for their actions and contribute ethically within group settings (24, 54). Furthermore, TPSR supports Goal 10 (Reduced Inequalities) but specifically 10.2, which promotes the social, economic, and political inclusion of all people, and 10.3, which seeks to ensure equal opportunities and reduce inequalities of outcome. Through structured reflection and guided practice, TPSR enables learners to recognise inequities, appreciate diversity, and act responsibly toward others.

#### 3.3.3 Sports education model

The Sports Education Model (SEM) was developed to provide students with authentic, educationally rich sporting experiences within the context PE (59). Its design seeks to replicate the structure and culture of sport as it exists in society, fostering meaningful participation and deeper learning through six defining features: seasons, team affiliation, formal competition, record keeping, culminating events, and a celebratory atmosphere (60).

A defining characteristic of SEM is its season-based organisation, which enables extended engagement with a smaller number of activities, thereby deepening students' understanding and skill development. Students of varied skill levels are assigned to teams that remain consistent throughout the season, fostering a sense of belonging, continuity, and shared responsibility. Within this stable structure, small-group activities enable students to plan, practise, and collaborate, enhancing both social interaction and cooperative learning (61). The competitive structure of SEM mirrors authentic sport experiences. Teams participate in scheduled games that contribute to a ranking system, promoting motivation and accountability.

According to Siedentop et al. (55), the overarching goal of SEM is to cultivate a learning context that generates experiences as authentic as possible to real sport. Beyond physical competence, the model provides opportunities for students to engage in diverse sport-related roles such as coach, referee, journalist, marketer, or team manager, thereby developing a broad range of transferable skills (55). These experiences strengthen key personal and social capacities including teamwork, leadership, empathy, responsibility, and autonomy (62). The SEM can also be interpreted through the lens of the Sustainable Development Goals (SDGs). It aligns closely with Goal 8.3, which advocates for the promotion of entrepreneurship, creativity, and innovation, as the model encourages students to assume initiative and demonstrate leadership within their teams (24). Furthermore, it supports Goal 8.2, which emphasises the pursuit of higher levels of economic productivity through diversification and innovation, and Goal 4.4, which focuses on equipping learners with skills relevant to employment and entrepreneurship. Through SEM, students can develop collaboration, communication, and organisational skills that extend beyond sport to professional and civic contexts.

Finally, SEM's emphasis on equity, cooperation, and shared decision-making reflects the values of Goal 16.7, which promotes inclusive and participatory processes. By integrating authentic sport structures with educational aims, the SEM can not only enriche the learning experience in PE but also contribute to students' personal development and the broader global agenda for sustainable and inclusive growth (24).

#### 3.3.4 Adventure learning model

The Adventure Learning Model (ALM) seeks to create learning experiences that engage students with the natural environment through activities involving elements of real or perceived risk (63). Rooted in experiential and environmental education traditions, this model is grounded in the belief that human potential is realised through meaningful interaction with nature. Adventure learning provides an educational context that challenges students physically and psychologically, fostering resilience, self-awareness, and environmental stewardship (64).

In PE, the ALM is particularly effective in promoting outdoor learning and physical activity during students' leisure time (64). By engaging learners in adventurous and exploratory experiences, it supports the development of problem-solving skills, cooperation, and emotional regulation. The WHO also highlights the importance of such approaches, recognising that participation in outdoor physical activities can promote environmental awareness and contribute to planetary health (65).

While environmental care is embedded within several SDGs, Goal 13 (Climate Action), Goal 14 (Life Below Water), and Goal 15 (Life on Land) have the most direct connections with PE. However, the ALM can be meaningfully aligned with Goal 13.1, which emphasises strengthening adaptive capacities to climate-related risks and natural disasters, and Goal 13.3, which advocates for improving education and institutional action to mitigate climate change (24). Adventure learning activities, such as orienteering, camping, or environmental expeditions, inherently build students' understanding of sustainability and climate resilience.

Additionally, the model aligns with Goal 12.1, which promotes sustainable consumption and the efficient use of natural resources, by encouraging learners to engage with and care for their environment responsibly. It also supports Goal 8.3, which highlights sustainable tourism and the promotion of local culture. Adventure-based learning can directly contribute to these aims by integrating physical activity with environmental appreciation and community engagement.

Empirical evidence further suggests that experiential learning in natural settings enhances students' global self-worth and perceived social acceptance (66). By combining physical challenge with reflective practice, adventure learning cultivates self-efficacy, collaboration, and ecological consciousness. Thus, within PE, the ALM not only enriches physical and social development but also contributes to global sustainability and wellbeing through transformative outdoor education.

#### 3.3.5 Independent production of teaching aids

The independent production of teaching aids involves the collection, recycling, processing and transformation of raw materials to produce teaching aids needed for PE teaching (67). However, educators should consider safety and liability implications when using independently produced materials. Teaching aids not purchased from certified manufacturers may not be covered under manufacturer liability policies; therefore, extra care should be taken to ensure such materials are safe, developmentally appropriate, and used correctly (e.g., routine inspection, clear usage protocols, and supervision). PE teachers believe that the use of self-made objects contributes to the cognitive, motor, physical, effective and value development of students (68). Furthermore, the creation of new resources for PE teaching by reusing materials encourages the development of ecological and responsible awareness, as well as personal values and attitudes that contribute to the achievement of these goals (69).

This model also appears in PE for other reasons. It appears as a solution to limited resources and the lack of sports equipment in many schools, it allows the creation of adaptable aids, increases students' motivation, enjoyment and interest, and the approach to this model allows for easy connection with other methodologies (70). In addition, previous research on self-made teaching aids has shown that this model allows for greater availability of props that help students increase their motor experiences in PE classes and increase exercise activity (71). Self-made teaching aids can motivate students to engage in physical activity outside of PE classes (72). In case students have difficulty implementing PE content in their free time due to economic barriers, self-made teaching aids could be a solution (68).

Furthermore, these ideas are in line with the Global Action Plan on Physical Activity 2018–2030 proposed by the WHO, highlighting the importance of promoting physical activity with an emphasis on environmental protection and sustainable development of the planet (15–17). This model could be linked to goals 13.1 and 13.3, which generally advocate the importance of a clean environment or reducing the consequences of global warming (24). There is also a connection between the model of self-production of teaching aids and global goal 12, or specific goals 12.1, which seeks to achieve sustainability in production and consumption processes, 12.2, which refers to the efficient use of natural resources, 12.5, which refers to the reduction of waste, and 12.8, which refers to ensuring the quality of information (24).

Finally, the collaborative environment that is created, as well as the creativity that is encouraged, will enable the development of skills related to entrepreneurship (goals 4.4 Improving skills for access to employment, decent work and entrepreneurship and 8.3 Entrepreneurship, creativity and innovation and promoting the formalization and growth of enterprises) (24).

### 3.4 Curriculum revision necessary for SDG alignment

To begin with, it is necessary to consider how societies define the purpose of education, which is a controversial and to some extent neglected issue (73). Every education system tries to fulfill a certain purpose (74). It is necessary to ask what this purpose is and how it affects the educational process. In this way, we can create new conditions, reexamine the existing situation and move beyond the usual criticism of PE (75). Lundvall and Fröberg (19) propose a critical analysis and revision of curricula and governing documents to determine which parts of the 2030 Agenda are applicable and appropriate in teacher education and PE in the context of education for sustainable development. The aim of the revision is to ensure knowledge-rich curricula that are competence-based. PE should be recognized as a core part of education, on an equal footing with subjects that teach reading and writing and subjects that develop mathematical skills. *Along with the arts, social studies, and health education, PE plays an important role in promoting civic education, democracy and human rights.* PE curricula should include objectives aimed at developing social competences, combining theoretical and practical knowledge based on research and best practices. Teaching methods should encourage discussion and exchange of information, with an emphasis on tolerance and understanding of diversity. PE and sport should be focused on lifelong learning, developing students' knowledge, skills and attitudes for active participation in society (85). Despite the clear pedagogical alignment between physical education and the SDGs, significant systemic barriers persist. Foremost among these is the ideological marginalization of the discipline, where deep-seated stereotypes often reduce PE to mere “gym class” or organized play, devoid of academic rigor (86). This cultural perception (87) directly influences fiscal policy, leading to frequent budget cuts, inadequate infrastructure, and a lack of professional development resources in both schools and universities (88). Furthermore, the academic workload prioritizes “core” subjects like mathematics and literacy, often at the expense of holistic disciplines (89). To genuinely advance the 2030 Agenda, curriculum reform must move beyond content updates to address these structural inequities, advocating for a shift in public and administrative consciousness that recognizes physical education as a critical driver of public health and social justice. PE objectives should be explored in different contexts, blurring the boundaries between the classroom and the gym, so that PE becomes an indispensable part of education for citizenship and civic values (76). Sustainability issues often have a whole-school approach (90). Taylor et al. (77) encourage each country to analyses its curriculum and initiate discussions on health and local action. Lundvall and Fröberg (19) also suggest that, through cooperation with schools, it should be determined which objectives are relevant at the local level.

According to Boström et al. (78), achieving sustainability-oriented education requires not only a multidisciplinary and holistic approach but also a fundamental shift in how learning itself is understood. To this end, they advocate for a transformative learning perspective, which Mezirow (79, p. 116) defines as “the process by which we transform problematic frames of reference (e.g., mindsets, habits of mind, perspectives of meaning), assumptions, and expectations to make them more inclusive, open, reflective, and emotionally capable of change”. This perspective invites learners to critically examine their own assumptions, beliefs, and emotions (83), and to reflect on how these shape their interpretations and actions (84).

Transformative learning reorients the learning process by fostering awareness of how individuals construct meaning. Within this pedagogical approach, teachers encourage students to interrogate the habits of mind and perspectives that inhibit change or perpetuate conflict across macro, meso, and micro levels (78). These levels reveal the interconnected influences of institutional structures, sociopolitical contexts, and power relations that should not be treated as isolated spheres of knowledge (82).

Adopting a transformative perspective allows for a deeper engagement with the structural and cultural barriers that constrain change, positioning the environment not merely as a passive backdrop but as an active dimension of learning (80). Through such an approach, students are better equipped to confront and respond to the complex challenges inherent in sustainability (81).

## 4 Conclusions

The SDGs form a global call to action under the United Nations' 2030 Agenda, aiming to create a fairer and more sustainable world. PE can play a meaningful role in advancing these goals. Through purposeful activities, students not only develop healthy habits but also acquire social skills that foster sustainable thinking and action.

While not all 169 SDG targets can be implemented through PE, this paper highlights that many are directly applicable. These can be addressed through many pedagogical approaches and strategies supported by over 20 years of research.

However, integrating sustainability principles into PE presents both challenges and opportunities to reimagine the field and universities must play a pivotal role in shaping the future of PE through the preparation of teachers who understand and apply sustainability principles.

Despite the obstacles also addressed in this paper, PE serves as a catalyst for transformation when its educational potential is fully recognized. By embedding sustainability in PE, educators can equip students with the knowledge, skills, and values needed to navigate and contribute to a rapidly changing world. This vision underscores the relevance of PE in advancing sustainable development and inspires optimism that meaningful change is possible.

## Data Availability

The raw data supporting the conclusions of this article will be made available by the authors, without undue reservation.
